# Implications of Circadian Rhythm in Stroke Occurrence: Certainties and Possibilities

**DOI:** 10.3390/brainsci11070865

**Published:** 2021-06-29

**Authors:** Dana Marieta Fodor, Monica Mihaela Marta, Lăcrămioara Perju-Dumbravă

**Affiliations:** 1 Department of Neuroscience, “Iuliu Haţieganu” University of Medicine and Pharmacy, 400012 Cluj-Napoca, Romania; lperjud@gmail.com; 2 Department of Medical Education, “Iuliu Hatieganu” University of Medicine and Pharmacy, 400202 Cluj-Napoca, Romania; mmarta@umfcluj.ro

**Keywords:** stroke, circadian rhythm, internal clock, chronotherapy, chronoprophylaxis, functional outcome

## Abstract

Stroke occurrence is not randomly distributed over time but has circadian rhythmicity with the highest frequency of onset in the morning hours. This specific temporal pattern is valid for all subtypes of cerebral infarction and intracerebral hemorrhage. It also correlates with the circadian variation of some exogenous factors such as orthostatic changes, physical activity, sleep-awake cycle, as well as with endogenous factors including dipping patterns of blood pressure, or morning prothrombotic and hypofibrinolytic states with underlying cyclic changes in the autonomous system and humoral activity. Since the internal clock is responsible for these circadian biological changes, its disruption may increase the risk of stroke occurrence and influence neuronal susceptibility to injury and neurorehabilitation. This review aims to summarize the literature data on the circadian variation of cerebrovascular events according to physiological, cellular, and molecular circadian changes, to survey the available information on the chronotherapy and chronoprophylaxis of stroke and its risk factors, as well as to discuss the less reviewed impact of the circadian rhythm in stroke onset on patient outcome and functional status after stroke.

## 1. Introduction

The temporal pattern of stroke occurrence could play an important role in treatment and prevention. Although seasonal and even circaseptan variations were described circadian variation is the most relevant in clinical practice.

## 2. Circadian Variation in Stroke Occurrence According to Etiopathogenetic Type

Studies on the circadian variation of stroke onset have been published since the 1970s, especially after the identification of circadian variation in the occurrence of cardiovascular events [1,2,3]. The first studies reported a higher frequency in stroke occurrence during the evening and night hours [4,5], but only a few recent findings support the night peak in stroke onset, especially in the case of lacunar ischemic events [6,7]. Since the 1980s, the results of most long-term studies consistently revealed a peak level incidence of stroke in the morning hours [8,9,10,11,12], this pattern is confirmed by the meta-analysis conducted by Elliott et al. [13]. Moreover, morning strokes occur more frequently soon after waking up, with 24% of all cerebrovascular events being found to take place within the first hour after waking up [8,14,15,16]. 

According to some opinions, the high incidence of cerebrovascular events in the morning is probably overestimated because it includes patients who wake up with stroke symptoms that began during night sleep but were only felt after waking up. Various researchers attempted to minimize this aspect either by considering that the stroke occurred during nocturnal sleep or by creating a group of so-called “wake-up stroke” patients for statistical analysis purposes. However, the pattern of circadian variation remained the same even after redistributing patients or separating the “wake-up stroke” cases, although the morning peak of stroke occurrence was no longer impressive [14,15,16,17,18,19].

Some retrospective studies on large groups of consecutive patients admitted over a few years reported a bimodal pattern of day-time variation with two peaks of stroke occurrence, a major one in the morning (6:00 a.m.–12:00 p.m.) and a less impressive, yet obvious one, in the late afternoon, between 6:00 and 8:00 p.m. [14,16,19,20]. This two-peak cycle (morning and afternoon-evening) was also reported by researchers from Mediterranean countries where the siesta is an integral part of the local lifestyle [21,22]. This suggests that the transition from sleep to wakefulness could represent an independent risk factor involved in stroke occurrence after a night sleep or siesta [19,23].

The circadian pattern of stroke occurrence proved to be generally similar, regardless of the etiology of stroke (Table 1).

Ischemic stroke (IS) has a major peak during the morning hours and a second minor one in the afternoon-evening period, even after adjustment by age, gender, vascular risk factors (hypertension, diabetes, hyperlipidemia, smoking habits), prior cerebrovascular or cardiovascular events, and previous treatment with antiplatelet or anticoagulant drugs [8,9,10,11,12,13,14,15,16,17,18,19,20,21,22,24]. Ischemic stroke subtypes (large artery atherosclerosis, lacunar, cardioembolic infarct—according to the TOAST classification) seem to have a similar circadian rhythm of occurrence [8,9,10,11,12,13,14,15,16,17,18,19,20,21,22,23,24]. However, some reports indicate that lacunar stroke has a higher frequency of occurrence between midnight and 6:00 a.m. [6,7,10]. Despite being different pathological entities with distinct pathophysiological mechanisms, both IS and intracerebral hemorrhage (ICH) proved to generally have the same cyclicity and common risk factors, especially the circadian variation of blood pressure (BP) values [8,20]. Nevertheless, reversed peaks were also reported, the afternoon-evening peak being more important than the morning peak, according to some authors [16,25]. Mortality was higher in patients with hemorrhagic stroke during the asleep versus the awake period, which was explained by the larger hemorrhage volume in the sleeping group [30]. Subarachnoid hemorrhage (SAH) does not fit the pattern of other types of strokes, the incidence of both aneurysmal and non-aneurysmal SAH being highest during the 10:00 a.m.–4:00 p.m. interval, while the cyclicity of the latter is rather controversial [8,26,27,28,29].

## 3. Triggering Factors

The circadian variation of cerebrovascular and cardiovascular events is similar as both share common triggering factors [2,3,19]. Their physiological circadian rhythms, especially rhythm alterations represent the main contributors to stroke occurrence, even more than the trigger itself [8,31,32].

### 3.1. Arterial Hypertension and the Dipping Profile

Blood pressure (BP), whose circadian rhythm is well known, represents the most studied risk factor for the occurrence of IS, by increasing the risk of fragile atherosclerotic plaque rupture, but also for the occurrence of hemorrhagic stroke by precipitating the rupture of the altered vascular wall [16,32,33]. The etiology of this circadian rhythm is based on exogenous factors such as the sleep-awake cycle, assuming the upright posture after waking up or the cyclicity of physical activity [8,14] as well as on endogenous factors influenced by daily rhythmicity, including autonomic nervous system activity, vascular tone modifications, and humoral factors (cortisol and catecholamine levels, or the renin-angiotensin-aldosterone system) [8,34,35]. However, various studies demonstrated that hypertensive and normotensive patients had the same chronobiological pattern of stroke occurrence, which suggests that the temporal variation of BP values, particularly changes in such values, contributed to this pattern more than arterial hypertension (AHT) alone [14,32,36]. Literature data also indicate a hereditary component in the circadian variation of BP and heart rate values, probably due to the hereditary character of the above-mentioned endogenous factors [37].

High BP values in the morning correlate with the highest morning peak of stroke occurrence [14,16,31,38]. Morning AHT occurs in two situations: night AHT continuing with morning AHT and the so-called “morning surge”. The first situation includes non-dipper patients with lower nocturnal fall in BP values and reverse-dipper patients whose nocturnal BP values are higher than the diurnal ones. The second situation is characterized by a sudden increase in BP values starting two hours before waking up and continuing afterward. Nonetheless, low BP values during the night (extreme-dipping) may contribute to the occurrence of night-time stroke, especially atherothrombotic infarction, lacunar infarction, and silent cerebral ischemia, via the hemodynamic mechanism and cerebral hypoperfusion [38,39]. On the other hand, the physiological circadian rhythm of BP values was frequently altered after stroke, with a shift towards the non-dipper or reverse-dipper patterns caused by damage to the autonomic nervous system resulting in sympathetic hyperactivity [35,40]. In conclusion, abnormal patterns in the circadian variation of BP (extreme-dipping, non-dipping, and reverse-dipping) represent independent predictive factors for stroke in hypertensive patients [18,31,38].

### 3.2. The Coagulation Balance

Circadian variability was observed in both the coagulation and the fibrinolytic systems, the morning hours being characterized by prothrombotic and hypofibrinolytic states. The increase in platelet aggregation and blood viscosity is especially seen on waking up and assuming the upright posture, probably due to an elevation in catecholamine levels, platelet, and red cell count in the first morning hours. [41,42]. An antiphasic circadian pattern was observed in the activity of the tissue plasminogen activator (tPA) involved in fibrinolysis, which has the lowest levels in the morning, associated with an increase in tPA 1 inhibitor, this combination having a hypofibrinolytic effect. This finding can have clinical significance since the effect of rt-PA treatment might be influenced by the circadian variation of hemostasis status [18,43]. An endothelial dysfunction was also reported in the same period, with higher common carotid artery intima-media thickness values and increased ubiquitin-proteasome activity which correlate with inflammation-induced plaque rupture [44,45].

## 4. Chronotherapy and Chronoprophylaxis

Data concerning the endogenous circadian cyclicity of the prothrombotic status, which peaks in the morning hours, as well as that of the profibrinolytic state, which is highest in the evening hours, may have clinical importance for the thrombolytic therapy of IS, although stroke units have reported different experiences. According to some authors, the effect of rt-PA infusion seemed to show a circadian variation in patients with ischemic stroke. IV thrombolysis with rt-PA appeared to be less effective when administered between 6:00 a.m. and 6:00 p.m. in comparison with the 6:00 p.m.–6:00 a.m. interval, while also being safer, with lower hemorrhagic transformation, in patients who started IV thrombolysis between noon and midnight [46]. A Chinese study on a similar number of patients did not show any difference between the four 6 h intervals of the day as far as IS onset or rt-PA infusion was concerned [47]. Other investigators found that patients with IS caused by middle cerebral artery occlusion had a better prognosis if thrombolysis was performed during day-time (9:00 a.m.–9:00 p.m.) compared with night-time (9:00 p.m.–9:00 a.m.) [43].

If the therapeutic window is missed, the main target is to save as much of the penumbra zone as possible, the neuroprotective treatment is one of the components in this approach. Information on the circadian variation of neuronal susceptibility to injury provided by experimental studies on rodent models of stroke revealed that neuroprotective drugs were more effective when administered during daytime (the inactive phase in rodents) compared to nighttime (the active phase), which corresponds to daytime in humans. A strategy targeting the administration of neuroprotectants in humans with acute stroke should be developed through translational studies and could explain why the neuroprotective agents effective in rodent models of stroke failed to provide neuroprotection in clinical trials [48].

Chronoprophylaxis could also be a mandatory approach in people at risk, by targeting the control of chronorisk factors through chronopharmaceutical medication. Chronopharmaceuticals are drugs administered at a particular time of day in order to control the expected onset of an event [49]. Hypertension, especially the morning rise in blood pressure may be the most important modifiable chronorisk factor able to reduce this peak. Thus, prescribing antihypertensive agents at bedtime for reducing the morning surge in blood pressure seems justified as it may prevent the morning peak of stroke occurrence. This approach should be individualized according to the dipping profile. It could prove beneficial for non-dipper and reverse-dipper patients, but care should be taken in the case of extreme-dipper patients in order to avoid an additional decrease in BP values and precipitate a night stroke through a hemodynamic mechanism, particularly in old people with carotid stenosis [31]. Therefore, this highly individualized approach requires outpatient blood pressure monitoring, especially in vascular risk patients [50]. The available evidence suggests that the main treatment for controlling the morning blood pressure surge is the use of long-acting drugs to cover the morning hours. Further studies are required to show that a reduction in BP morning rise, through a chronopharmaceutical approach, might improve stroke prevention [51].

Recent data suggest that a low dose of aspirin administered at bedtime decreased COX-1 dependent platelet function in the morning in healthy and cardiovascular patients, thus having a beneficial anti-aggregation effect during the vulnerable morning period compared with administration in the morning hours [52,53]. Moreover, 100 mg of aspirin taken in the evening reduced plasma renin activity and the excretion of cortisol, dopamine, and norepinephrine in the 24 h urine of grade 1 hypertensive patients compared with administration in the morning, which demonstrated a BP-lowering effect [54], although these results were not validated by studies on larger numbers of patients [52].

The anticoagulant effect of rivaroxaban, a direct inhibitor of activated factor X, taken at bedtime was found to better control morning hypofibrinolysis [55].

The treatment of sleep disorders could potentially have a protective role in stroke prevention. Melatonin for the treatment of insomnia not only corrects sleep disturbances but also helps resynchronize the internal clock, in this way restoring the natural biorhythm, possibly improving stroke prevention, favoring neurorehabilitation, and increasing brain-derived neurotrophic factor (BDNF) expression [56]. Randomized studies are needed to assess the therapeutic efficacy of CPAP (continuous positive airway pressure) for stroke prevention in patients/ individuals with obstructive sleep apnea [57].

The chronotherapeutical approach involves more than prescribing medication at a certain hour or according to circadian behavioral patterns, i.e., before bedtime or after waking up, before or after meals. It also considers the endogenous circadian rhythm, which may vary depending on age, comorbidities, or behavioral cyclicity. The circadian phase can be assessed using serial salivary melatonin assays. The development of a less expensive and more practical biomarker of the internal circadian phase would be highly beneficial [58]. This could allow prescribing medication according to specific circadian phases. Resynchronization of the internal clock when important changes or delays occur would be a breakthrough. There are limited options for the pharmacological resynchronization of the internal clock, melatonin administration being the most convenient choice. Experimental studies on the treatment and prevention of stroke have opened new directions like the use of novel astrocyte-specific targets through pharmacological methods or gene therapy in order to manipulate the clock mechanism and/or modulate neuronal susceptibility to injury [57]. Conversely, simple non-pharmacological approaches like regulating exposure to light and dark, routine physical activity, and temporary fasting could enhance clock function, reduce vascular risk factors such as diabetes, obesity or sedentarism, and prevent stroke occurrence [59].

## 5. Impact of Circadian Variation in Stroke Onset on Outcome and Functional Status

The literature provides limited data on the role of the circadian moment of stroke occurrence on patient outcome after hospital discharge, especially on the subsequent evolution in time and the degree of rehabilitation.

The retrospective study conducted by Ripamonti et al. on 3689 patients admitted to a stroke unit over a period of 10 years suggested that the time of stroke occurrence may correlate with prognosis and outcome, measured by the modified Rankin scale (mRs) on hospital discharge and by 30-day mortality. The IS patients with asleep onset had worse mRS values on hospital discharge and higher 30-day mortality compared to those with awake onset, in agreement with the findings of Jimenez-Conde et al., who found that initial stroke severity measured by the NIHSS (National Institutes of Health Stroke Scale) was higher in nocturnal stroke onset, but without differences in functional status at 3 months compared with the diurnal onset of IS [20,60]. The better disability scores recorded in awake IS patients were influenced by the 10% who met the criteria for thrombolysis [20]. Liou et al. reported slightly different results regarding the timing of IS onset on functional status. They focused on evaluating the disability of IS patients on discharge using the mRS scale and the Barthel Index (BI), the latter being more detailed and self-care-oriented. While the disability degree measured by the BI did not show significant differences between the six 4 h intervals of the day used to determine stroke onset, the disability measured by the mRS revealed that the best functional status was obtained if the stroke occurred within the 4:00 a.m.–8:00 a.m. interval, while the worst results were reported for occurrence between 8:00 p.m. and 12:00 a.m. These intervals overlap the transition from asleep to awake and vice versa [61]. Shokri et al. reported a lower NIHSS and longer onset-to-door time if the event occurred during the afternoon interval, but the outcome measured by mRS at 3 months did not vary according to onset time [62].

There are different data concerning the disability of ICH patients on hospital discharge: some authors reported the worst mRS values in patients who experienced ICH during the awake period [20] while others reported a higher disability for an occurrence during sleep, because of the larger hemorrhage volume in asleep patients [30]. According to a multicenter study carried out by Fabio et al., ICH patients reporting to the emergency department in the morning (6:00 a.m.–12:00 p.m.) and during the night (12:00 a.m.–6:00 a.m.) had higher intra-hospital mortality than those who arrived in the afternoon interval (12:00 p.m.–6:00 p.m.) [63].

The evolution of the severity of the clinical picture, as well as the disability and cognitive status, were assessed for a longer interval after stroke using the NIHSS, mRS, and MMSE (Mini-Mental State Evaluation) in only a small group of 63 patients with IS who were followed-up for two years in a rehabilitation center. Thus, Fodor et al. found that the patients with nocturnal onset of IS had the worst evolution according to all three scores in the second year after the cerebral ischemic event, which indicates poor prognosis compared to the patients with IS onset during the other intervals [64,65].

## 6. The Internal Clock and Its Possible Implications for the Occurrence and Severity of Cerebrovascular Events

The system that maintains the circadian rhythm has a complex cytoarchitecture with two poles: the master clock located in the suprachiasmatic nuclei (SCN) of the bilateral ventral hypothalamus, which is synchronized with the geophysical time through changes in light intensity, and subsidiary clocks in almost all body cells. The molecular basis of these “clocks” consists of a transcription-translation feedback loop made of BMAL1-CLOCK/NPAS2 activators (“the positive limb”) which heterodimerize, bind E-box motifs, and modulate the transcription of a multitude of genes, including the circadian repressors (“the negative limb”) represented by period (PER1–3) and cryptochrome (CRY1 and 2), which inhibit the transcription of the “positive limb”. This clock mechanism also exists in neurons and astrocytes, as well as in almost all body cells. Clock disturbances or desynchronization lead to subsequent molecular, cellular, and physiological alterations, which may influence the risk of stroke occurrence, the susceptibility to injury, and the recovery capacity of the nervous tissue [66,67,68]. Elucidating this biological mechanism may offer solutions for stroke prevention and treatment.

### 6.1. Circadian Rhythm in Neuronal Susceptibility to Injury

Recent data suggest the existence of circadian variation in the neuronal susceptibility to injury of the various brain regions, which means that the extent of cerebral damage depends on when lesions occur. Although this is difficult to prove clinically, the results of experimental studies on rats with induced global cerebral ischemia via hemodynamic mechanisms (through transient cardiac arrest) at various moments throughout the day revealed that the effects of cerebral ischemia measured by the level of caspases−3 and −9 for the intrinsic apoptotic pathway (mitochondrial) and of caspases−8 for the extrinsic pathway (receptor-mediated) were more severe if the event occurred during active hours (especially the first hours) compared to inactive hours. Moreover, in rats with experimentally-induced focal ischemia, the volume of cerebral infarction was up to three times larger and the penumbra zone was narrower during the active period with high susceptibility compared to the inactive period [48,50].

### 6.2. Circadian Rhythm in Astrocyte Activity

Although research on cerebral ischemia mainly focused on neurons, recent studies also explored astrocytes, which represent an important non-neuronal cell population in cerebral tissue that supports neurons and whose activity may have either neuroprotective or neurotoxic effects in case of ischemic injury. While neurons use blood glucose as the only source of energy for producing adenosine triphosphate (ATP), astrocytes also employ alternative sources such as glycogen reserves, which are transformed into glucose and lactose for ATP synthesis and then delivered to neurons when needed. Thus, neurons are more susceptible to an acute fall in glucose during ischemic stroke, decreased ATP production being a potential cause of neuronal death. However, astrocytes can partially support neurons by releasing ATP near synapses, where it is converted to adenosine, which reduces excitotoxicity and displays a neuroprotective effect. The data suggest that the production of ATP by astrocytes has a circadian rhythm [58,69]. A circadian rhythm was also noted in the astrocyte regulation of the uptake and release of excitotoxic glutamate in cerebral tissue, with a peak in extracellular glutamate in the second half of the day in mice, when ischemia produced more neuronal damage. The same temporal pattern of fluctuation occurs in the response to oxidative stress and astrogliosis. [58,70]. These findings lead the way to developing new approaches that target not only neuronal but also astrocyte cell populations.

### 6.3. Desynchronization of the Endogenous Clock

Different causes may lead to the desynchronization of the internal clock relative to the terrestrial rhythm, particularly when individuals choose to follow circadian patterns that fail to overlap the endogenous clock. Deeper changes in the inner clock occur in sleep disorders and aging. Various organic brain lesions will lead to the disruption of the clock mechanism.

#### 6.3.1. Sleep Disorders and Stroke

Sleep disturbances (obstructive sleep apnea, insomnia, short/long duration of sleep, and sleep-related movement disorders such as periodic limb movements during sleep or restless legs syndrome) represent risk factors for developing stroke and cardiovascular events, precipitated by circadian desynchronization [71].

Large cohort studies conducted in different countries revealed a high incidence of cerebrovascular and cardiovascular events in people with sleep disturbances [56,72,73]. Although the exact mechanism by which sleep disorders precipitate stroke occurrence is not clear, sleep is known to interfere with neuroplasticity, thus having an important neurorestorative role. Stroke itself can cause different sleep disorders, desynchronizing the activity of the central nervous system and impairing the master clock or its connections. The persistence of circadian rhythm desynchronization and consequently of sleep disturbances can negatively impact rehabilitation after stroke [56].

#### 6.3.2. Age-Related Desynchronization of the Internal Clock

Aging of the nervous tissue decreases the expression of core clock proteins leading to the dysregulation of the endogenous clock in neurons and glia. The age-related changes in the internal clock include a decrease of the amplitude and robustness of rhythms or even in fragmentation or loss of rhythms. Impairments of the positive limb of the circadian clock in the brain, produced by deletion of either Bmal1 or Npas2 and Clock induce widespread astrogliosis, neuronal oxidative stress, and axonal terminal degeneration, thus highlighting the implications of the circadian clock positive-limb transcriptional complex (BMAL1: CLOCK/NPAS2) in the protection against neurodegeneration and in neuron susceptibility to injury, including ischemia [50,67,74].

#### 6.3.3. Exogenously Generated Light-Dark Cycle

Exposure to artificial light (which is now possible at any time during the day), shift work, jet lag, and various lifestyle options (night-time working, eating, or engaging in various social activities) may cause repeated endogenous circadian clock changes that can have a negative impact on human life. The consequences include oxidative stress, inflammation (an increase in inflammatory markers including IL-6, C-reactive protein, and TNF-α), and loss of synapses [75,76,77].

#### 6.3.4. Desynchronization of the Internal Clock after Stroke

While dysregulation of the internal clock due to different causes may lead to stroke, the stroke itself produces disruption in the circadian endogenous rhythm by directly affecting the STNs or their connections and disturbing the neuronal clock mechanism [50,56]. The data show that neuronal ischemia produced advanced PER1 expression [35] and induced changes in the timing of pineal melatonin secretion, which regulates the expression of Bmal1, resulting in its decreased expression, both melatonin and Bmal1 playing a critical role in cell survival in neuronal ischemia [50,78,79]. In SAH, a suppressed expression of PER1 was found in CSF, correlated with short-term mortality and the extent of delayed ischemia in the case of vasospasm [80].

Clinically, dysregulation of the internal clock leads to imbalances in the sleep-awake cycle, hence causing sleep and mood disorders, altered circadian variation of physiological BP values, and a shift towards pathological dipping profiles (non-dipper and reverse-dipper). These consequences can have negative long-term effects on recovery after stroke and create a vicious cycle that predisposes to repeated strokes [34,56,63].

## 7. Conclusions

Knowing that the highest incidence of stroke occurs during the morning hours represents valuable medical and sociological information that can impact the admission, evaluation, and treatment of stroke patients in emergency or stroke units by raising a red flag to ensure the availability of adequate human and material resources during this interval (service on demand). Moreover, this evidence opens new perspectives in the chronotherapy and chronoprophylaxis of cerebrovascular disease. Preventive strategies must focus on the risk factors of stroke, including their circadian variation and internal clock resynchronization through pharmacological and non-pharmacological approaches. To our knowledge, this is the first review on the impact of the timing of stroke onset on functional status after stroke, which may be important for predicting recovery and estimating the medical and social resources involved in the rehabilitation process.

## Figures and Tables

**Table 1:** Circadian variation in stroke occurrence according to stroke type.

Stroke Type		Circadian Pattern of Occurrence	References
IS	LAA	Major peak in the morning ± minor peak in the afternoon	[8,9,14,19,22,24,25]
	Cardioembolism	Major peak in the morning ± minor peak in the afternoon	[8,9,14,19,20,21,24]
	Lacunar stroke	Morning peak or night peak	[8,9,14,19,20,24] [6,7,10]
ICH		Morning peak or afternoon peak	[8,20] [16,25]
SAH		Higher occurrence between 10:00 a.m.–4:00 p.m.	[8,26,27,28,29]

IS—ischemic stroke, LAA—large artery atherosclerosis, ICH—intracerebral hemorrhage, SAH—subarachnoid hemorrhage.
